# A Novel Approach to Treat Traumatized Alveolar Ridges: Two Case Reports

**DOI:** 10.1155/2016/9312412

**Published:** 2016-08-09

**Authors:** Mahesh Verma, Sneha Menghani, Jyoti Devi, Rekha Gupta, Shubhra Gill

**Affiliations:** Department of Prosthodontics, Maulana Azad Institute of Dental Sciences, New Delhi, India

## Abstract

Functional forces are transmitted to the basal seat mucosa through a hard denture base during mastication. Such hard base dentures are not comfortably tolerated in patients with fragile oral mucosa and will cause sore spots, masticatory pain, and further resorption of alveolar bone. Soft liners materials can be advocated successfully to manage such clinical situations. The soft liner material absorbs masticatory forces by means of the cushioning effect and distributes occlusal forces uniformly to prevent trauma to compromised residual ridges.

## 1. Introduction

Edentulous patients often seek prosthodontic care to replace their missing teeth for improvement in esthetics, function, and speech [1].

In India, average life expectancy, which used to be around 42 in 1960s, has steadily climbed from 48 yrs in 1980 and 58.5 yrs in 1990 to around 62 yrs in 2000. The overall health indicators have also shown significant improvement across the country in the past decade. According to the latest WHO data the numbers have increased to 67.3 (2011–15) amongst men and 69.3 (2011–15) amongst women [2]. With the increase in survival at older age there is an increase in the numbers of patients who seek treatment for edentulism. Conventional removable complete dentures fabricated using PMMA based heat cure resins are most commonly used for prosthodontic rehabilitation of completely edentulous patients. However, some patients are maladaptive in their ability to tolerate conventional removable prosthodontic treatment and might require more advanced treatments [3].

The implant-supported prostheses have vastly improved the final outcome related to treating edentulous patients [4]. However, this treatment modality might not be possible in all completely edentulous patients due to financial constraints, inadequate bone quantity and quality, and unwillingness of the patient to undergo a surgical procedure. Therefore, fabrication of conventional dentures is the mainstay of treatment for many of these patients.

In conditions with grossly resorbed ridges, knife edge ridges, or ridges with severe undercuts, depressed irregular ridges, conventional dentures have compromised retention and they continuously cause trauma to the mucosa covering the undercuts during insertion and removal of the prosthesis, thereby reducing patient compliance. Comfort in these conditions can be improved by relining the hard denture base of complete dentures. Other indications for relining include residual ridges with thin, sensitive mucosa, flabby tissue, and congenital or postoperative defect areas restored with obturators [5].

Incorporating a layer of resilient permanent soft liner within the denture base improves retention in atrophic flat ridges with inadequate vestibular depth by intimate contact with underlying tissues and also reduce the traumatic impact to residual ridges by distributing masticatory load. The resilient layer acts as a shock absorber by absorbing some of the load and equal distribution of remaining stresses during function, so that the hard basal seat of the denture receives less impact force [6].

Permanent silicone soft liner Molloplast B (Detax GmbH and Co., West Germany) supplied as a one-paste system consists of a polymer (polydimethylsiloxane), cross-linking agent (acryloxyalkylsilane), and catalyst (heat and benzoyl peroxide). Adhesive (gamma-methacryloxypropyltrimethoxysilane), which is a silicone polymer, acts as a solvent to aid bonding to the denture base.

Molloplast B retains viscoelasticity and softness for longer periods and does not harden due to lack of plasticizer. Being elastic in character, it is stretched during insertion and removal of prosthesis over bony prominences without traumatizing the tissues and springs back into close contact with the undercut area thereby improving the retention.

The current case reports describe a novel and reliable method of relining to treat traumatized alveolar ridges where any other technique has failed to provide relief to the patient. First case report shows the incorporation of silicone liner at the time of lab processing of denture which resulted in improved bonding of permanent reline material with the PMMA resin also reduced patient's time utilized in comparison to chairside relining. And the second case report shows the application of soft liner after 1 year of the fabrication of the denture, when patient continued to have pain even after multiple adjustments in the depressed undercut areas of the ridge where mucosa was thin and fragile.

## 2. Case Report 1

**Figure 1:**
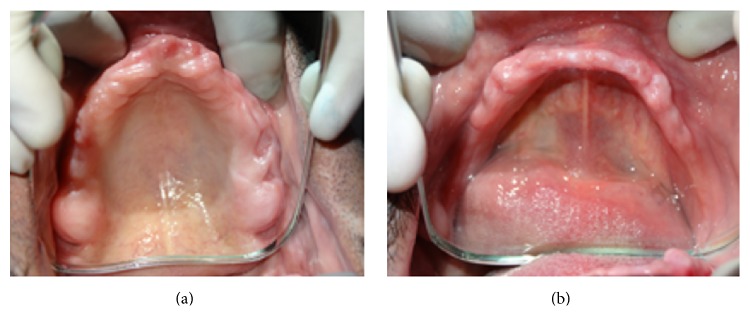
Unhealed maxillary and mandibular residual ridges with severe undercuts.

A 42-year-old male reported to the outpatient Department of Prosthodontics and Crown and Bridge, Maulana Azad Institute of Dental Sciences, New Delhi, with the chief complaint of inability to wear dentures for longer duration and associated soreness of jaw bone. A preliminary intraoral examination revealed completely edentulous arches with generalized erythema of the mucous membrane of the upper and the lower residual ridge (Figures 1(a) and 1(b)). The extraction sockets were unhealed even after 8 months of extraction. Medical history revealed that the patient was diabetic and was on oral hypoglycaemic drugs since 5 years. Refabrication of a new set of complete dentures relined with a silicone based heat cure liner was planned for the patient.

### 2.1. Procedure

#### 2.1.1. Clinical Steps

**Figure 2:**
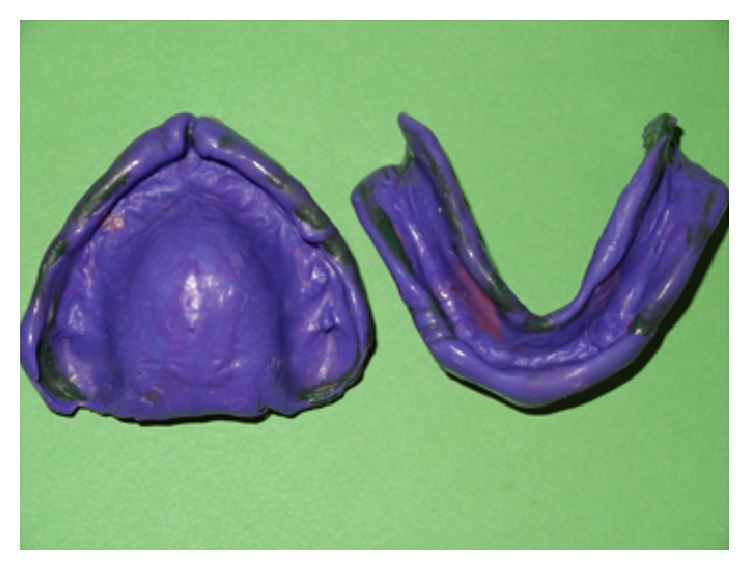
Maxillary and mandibular final impression made using elastomeric impression material.

Maxillary and mandibular primary impressions were made using irreversible hydrocolloid. The special tray was constructed using autopolymerizing resin on the primary cast thus obtained. Border molding was done using green stick compound and a wash impression was made using elastomeric impression material due to the presence of undercuts (Figure 2). On the secondary casts obtained, clinical steps of jaw relations and try-in were completed in the conventional manner.

#### 2.1.2. Laboratory Steps

**Figure 3:**
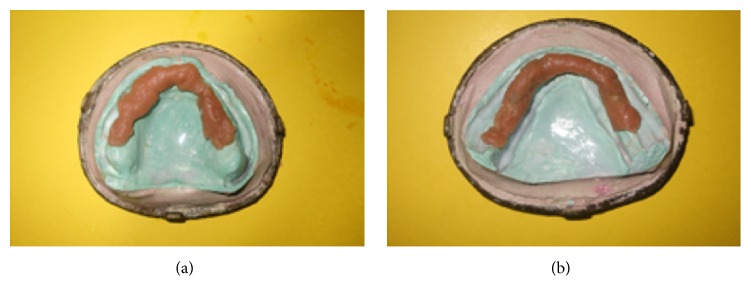
Permanent heat cure silicone liner (Molloplast B) adapted over maxillary and mandibular residual ridge crest.

**Figure 4:**
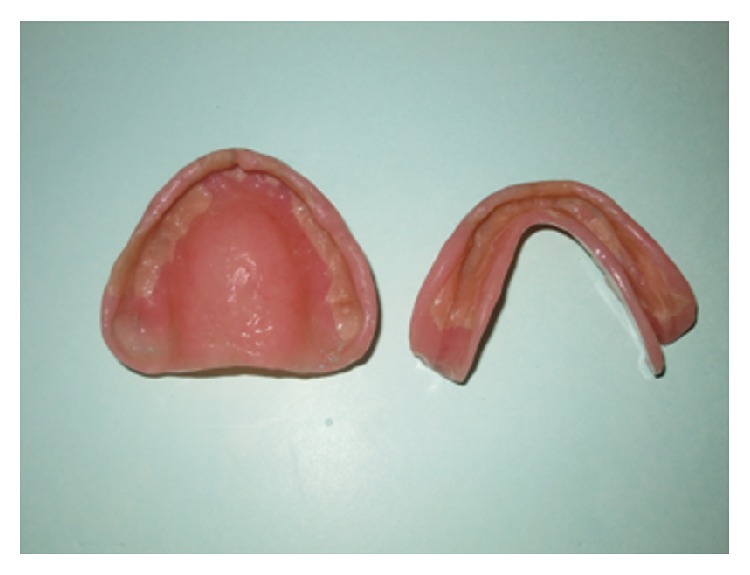
Intaglio surface of relined dentures.

The final waxed up dentures were invested. After wax elimination, separating agent was applied before packing. Permanent heat cure silicone based soft liner (Molloplast B, DFS) was adapted on the crest of the residual alveolar ridges (Figures 3(a) and 3(b)). Heat cure based acrylic resin was packed in doughy consistency and trial closure was done using cellophane sheet. On opening the flask, any soft liner material reaching to the denture border was cut and acrylic was filled. The packed dentures were cured, finished, and polished (Figure 4). Polishing of acrylic was done using conventional acrylic finishing burs (SHOFU). The functional borders of relined denture was trimmed with Molloplast® special cutters or grinding sleeves (15–20 thousand r.p.m.), without problems and heat build-up. Molloplast prepolishing discs were used for smoothing of rough spots.

The dentures were inserted and adjusted. Postinsertion instructions were the same as given for any removable prosthesis. Follow-up was done at intervals of 6 months for 2 years. The patient had adapted well to the dentures without complains of sore mouth. The mucous membrane overlying the residual alveolar bone was firm, resilient without signs of inflammation. The soft liner material showed no signs of tear or staining.

## 3. Case Report 2

**Figure 5:**
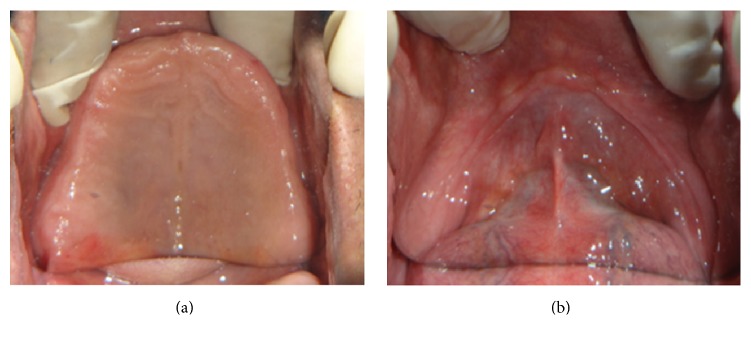
Maxillary arch and mandibular severely resorbed irregular residual ridge with elevations and depressions.

A 47-year-old male reported to the outpatient Department of Prosthodontics and Crown and Bridge, Maulana Azad Institute of Dental Sciences, New Delhi, with the chief complaint of difficulty in chewing food due to loss of teeth 5 years ago. History revealed that reason for loss of teeth was aggressive periodontitis with 36 and 46 teeth being removed at a very young age of 35. History of bidi smoking was also present for 10–15 years but he left the habit 5 years ago after the loss of teeth. He was a first-time denture wearer. Intraoral examination revealed completely edentulous arches with severely resorbed lower residual ridge with body prominence in the mid symphyseal region and depressed areas with respect to 36 and 46 region (Figures 5(a) and 5(b)). Medical history did not reveal any significant findings. Fabrication of complete dentures was planned for the patient.

### 3.1. Procedure

#### 3.1.1. Clinical Steps

**Figure 6:**
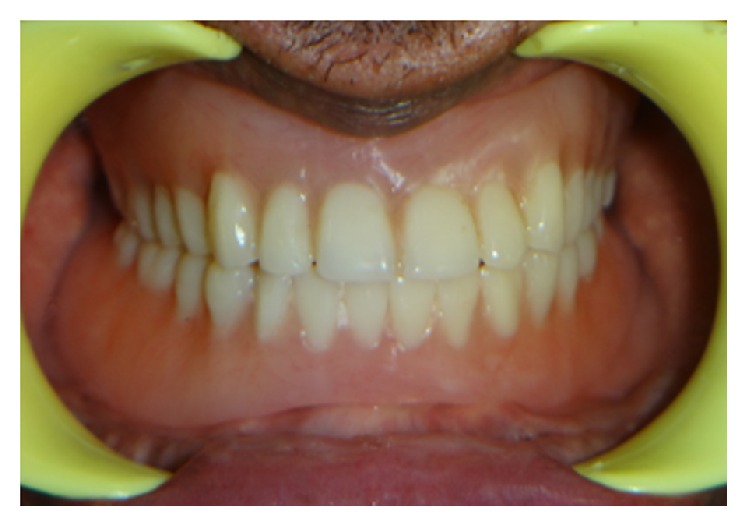
Maxillary and mandibular dentures delivered conventionally.

Maxillary and mandibular complete dentures were fabricated in the conventional manner (Figure 6). Selective pressure impression technique was used for mandibular final impression and semianatomic posterior teeth were used to reduce the stresses on the edentulous ridge at the time of try-in.

Follow-up was done after 24 hours of denture insertion and further after 3 weeks and 3 months. Even after 3 months of denture wear with all the instructions followed by the patient, he could not adjust with his new dentures with complains of sore mouth and ulceration along with pain specially in the elevated midline area and depressed 36 and 46 regions although occlusion was satisfactory. Pressure indicating paste was used in 2 consecutive sittings which could provide only temporary relief for few days. After 2 months of adjustment, patient then reported after a gap of 6 months again with the same problem in the same areas. Adjustment was done again and patient remained asymptomatic for the next 4 months. Thus, when it was seen that even after 1 year of denture wear patient is not fully satisfied, going for permanent soft reline was decided as the mucosa in the symptomatic areas was fragile, thin, and not firmly attached to the periosteum. Patient did not have any other problem or difficulty with the dentures while chewing.

**Figure 7:**
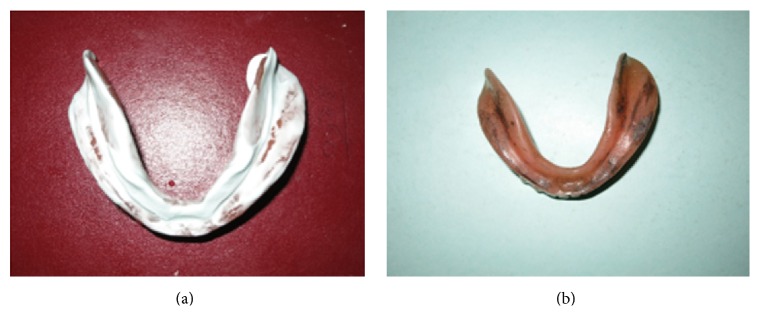
Pressure areas detected with pressure indicating paste and marked with pencil.

**Figure 8:**
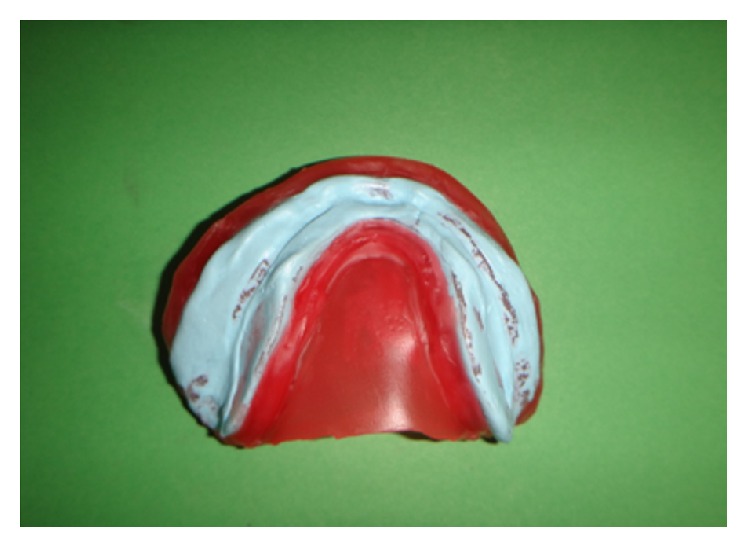
Beaded wash impression made with noneugenol zinc oxide paste.

Pressure indicating paste (COLTENE) revealed pressure spots in the anterior, 36 and 46 region (Figures 7(a) and 7(b)), which were marked with a pencil and trimmed till the time patient was fully asymptomatic. A wash impression was made using noneugenol based zinc oxide paste (CAVEX) (Figure 8) after making a uniform space of 1 mm approx. for the wash impression.

#### 3.1.2. Laboratory Steps

**Figure 9:**
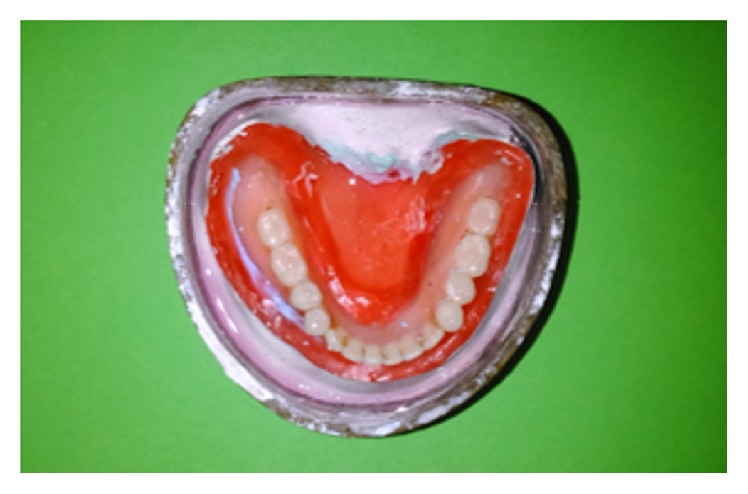
Invested mandibular denture.

Beading and boxing of impression were done; new master cast was poured in dental stone and directly invested (Figure 9). Dewaxing was done, and denture was cleaned thoroughly using the steam cleaner and intraoral sandblaster. After that 1.5 mm of uniform reduction was done on the tissue surface to accommodate the silicone liner. Uniform holes of 1 mm diameter were made on the undersurface using a carbide round end bur to improve the surface area for bonding of the liner.

**Figure 10:**
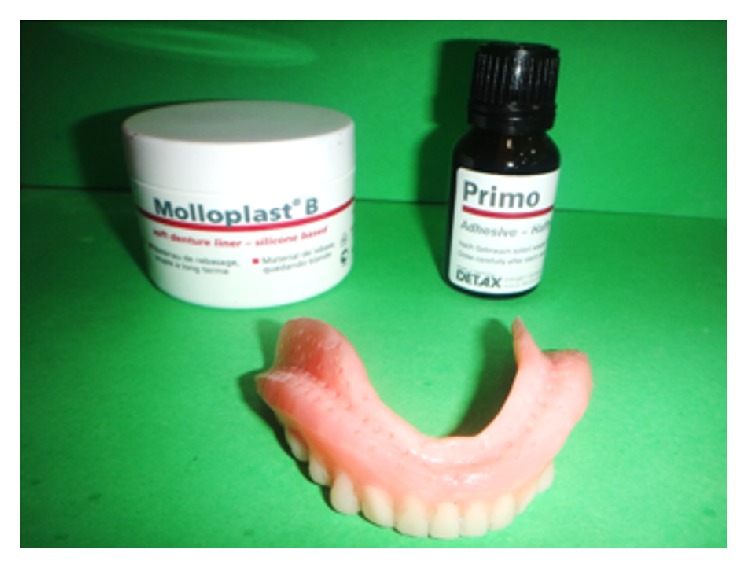
Adhesive applied on the tissue surface after making holes 1 mm in diameter 2-3 mm apart from each other.

Primer/adhesive was applied on the surface and was allowed to dry (Figure 10). After that packing of the silicone liner was done in the similar manner as done for heat cure resin. Flask was placed in cold water and slowly heated up to 100°C and polymerisation in boiling water continued for approx. 2 hours. Flask was allowed to cool down slowly taking care not to “shock-treat” it by rinsing with cold water immediately. Denture was retrieved, finished, and polished following the same steps as discussed in the first case report.

**Figure 11:**
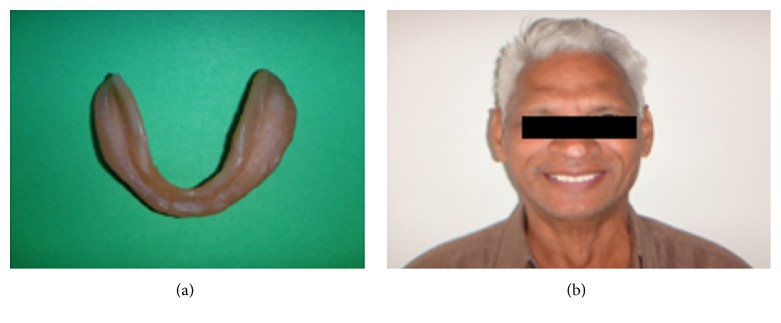
Relined mandibular denture and pain-free satisfied patient.

The relined lower denture was inserted and was checked for any pain or discomfort. Adjustments were done and postinsertion instructions were given (Figures 11(a) and 11(b)). After the patient became fully asymptomatic, follow-up was done at intervals of 6 months for 1 year. The patient had adapted well to the relined dentures without complains of sore mouth as it was before. The mucous membrane overlying the residual alveolar was healthy without the signs of any inflammation or soreness. The soft liner material was also soft and did not show any sign of tear or detachment.

## 4. Discussion

The greatest virtue of soft liners lies in their versatility and ease of use. In a 6-year retrospective study, 93% of the edentulous patients felt more comfortable when the denture was lined with a soft liner [7].

Clinical trials also demonstrated that the application of soft denture liners to mandibular complete dentures improved masticatory ability of edentulous patients and decreases discomfort during the first adjustment [8, 9]. Another retrospective investigation conducted into the serviceability of Molloplast B-lined dentures concluded that Molloplast B is not a temporary expedient but rather can remain serviceable for a time competitive with that of conventional acrylic resin dentures. It is available as a ready to use single component silicone, eliminating mixing and dosing error. It stays soft permanently and features decades of documented success in denture relines. The material bonds firmly to new or existing acrylic dentures and stays elastic and bacteria-free for years.

It can be polymerized simultaneously with acrylate when making new dentures but can also be used for relining of already-worn dentures. The longevity of the soft liner is dependent on correct processing procedures and proper home care. The resilience of the liner is dependent on its thickness and the optimum thickness is approximately 3 mm. The resiliency of the liner did not decrease with time and no noticeable wear of the soft liner was observed. Staining was present in about half the soft liners, which was commonly associated with habits like smoking [10].

Despite all the advantages special care has to be taken regarding maintenance of excellent oral and denture hygiene when using soft liners, as there are chances of fungal growth on the soft liner material. Use of denture cleansers with antimicrobial agents is generally recommended. Also, the functional duration of permanent soft liner is short and requires frequent follow-up to check for tear of soft liner. Entire gingival margin of remaining teeth should be covered or else can lead to plaque accumulation.

## 5. Conclusions

Relining of new or old dentures which are otherwise functioning well with permanent silicone soft liner is a novel approach to minimize trauma to compromised denture-supporting tissues and thin mucous membrane overlying prominent bony prominence and also improves the retention of the prosthesis by maintaining intimate contact with the tissues.
